# Remembering past emotions: How emotion expressions are linked to memory reappraisal

**DOI:** 10.1371/journal.pone.0332575

**Published:** 2025-09-24

**Authors:** Deniece S. Nazareth, Khiet P. Truong, Dirk Heylen, Peter Kok, Gerben J. Westerhof

**Affiliations:** 1 Psychology, Health and Technology, University of Twente, Enschede, The Netherlands; 2 Human Media Interaction, University of Twente, Enschede, The Netherlands; 3 Netherlands eScience Center, Amsterdam, The Netherlands; University of Lahore - Raiwind Road Campus: The University of Lahore, PAKISTAN

## Abstract

This paper examined how older adults experience the past and present valence of emotional memories and its relation to their multimodal expressions. As older adults can become more biased towards positive emotions and minimize negative emotions, a valence difference in the reappraisal of memories can exist. A study was conducted with older adults (N = 17) who self-reported their past and present valence of emotional memories. Data were collected between July and November 2018. Using automatic recognition technology, facial, acoustic, and lexical expressions were analysed during memory reminiscence. Results showed that differences exist between the past and present valence. Due to the reappraisal of the memory, a shift in valence occurred as a strong fading effect was seen in the sad memories, while a slight positive bias was also present as the older adults were more biased towards positive emotions in happy memories. Different modalities were more relevant for past than for present valence. These findings show that distinguishing between past and present valence is important when studying emotions in older adults’ memories. Older adults are more biased towards positive emotions and minimize negative emotions. Finally, automatic emotion recognition technology can benefit from these findings to assist older adults in the future.

## Introduction

In the current aging society, it is important to gain a better understanding of how older adults are feeling in order to support them in maintaining or improving their quality of life, independence and physical care [1]. By understanding their emotional experiences, we can anticipate and adapt to their needs. For older adults, reminiscing about memories is a very natural process to regulate their emotions [2,3]. As age also impacts the expressions of emotional experiences, it is interesting to examine how this is conveyed in different modalities such as face, voice, or words. Age-related changes in modalities can pose a challenge for the accurate use of automatic recognition technology for older adults. Age-related changes in the face, such as folds and wrinkles, may suggest a certain emotional expression [4]. Other facial features often found in older adults, for example the mouth corners that are turned down, can be misinterpreted and decoded as a negative emotional expression [5]. For the lexical modality, Pennebaker and Stone [6] found in their research on age and language use that, with increasing age, people use fewer negative and more positive affect words.

In this paper, the goal is to examine how multimodal expressions are related to the valence of emotional memories recalled by older adults by using automatic recognition technology. Although the complexity and impact of aging on expression make the applicability of this technology challenging, it also allows us to study emotional expressions of older adults on a larger scale in a consistent manner.

### The experience of emotional memories

Older adults often reminisce about past memories as it is a natural process for them [7]. Recalling a specific memory tends to reinstate the associated emotions of a specific past experience [8]. This implies that older adults can re-experience their past emotions in their memories during reminiscence. However, studies on personal reminiscing have found that older adults showed positive biases during recall and experienced more positively and less negatively evaluated emotions [9–11].

This difference in valence can be explained in how people reappraise past memories or events. According to the appraisal theory, people re-evaluate their feelings by appraising the emotional memory in the present [12,13]. Here, appraisal can be defined as event evaluations that reflect the relation of an individual’s goal with that event. Events seen as goal fulfilling elicit positive emotions, whereas goal hindering events are associated with negative emotions. Thus, these remembered emotions are based on a person’s present appraisal of the importance of a goal [14]. Emotions remain consistent, given that goals remain unchanged. With time however, the importance of goals can change as people are gaining other ambitions, coping with new events, or are minimizing the importance of them in order to behold certain goals [15,16]. In addition, the perspective of time can also lead to a change in goals [17]. When people age, they become more aware of their limited time [18] and their goals shift from future knowledge-related goals to more short-term present goals, such as emotion regulation and social support that is emotionally gratifying [17,19,20]. This prioritization by older adults leads to a change in the recall and reappraisal of their memories, which in turn impacts the current emotion. A large difference in the reappraisal will result in a greater difference between past and present emotions [13]. In their shift towards emotional gratifying goals, older adults will more likely maximize positive emotions and minimize negative emotions [19].

These biases are also known as the positivity bias and the fading affect bias that are often present in older adults. These biases grow stronger over time [21]. Multiple studies show that older adults are more positively biased in their emotional experiences and expressions [10,22]. They are biased towards positive emotions and recall past memories more positively [23] while they react less to events that induce negative emotions or even ignore these emotions [24,25].

According to Walker and Skowronski [26], the goal of the positivity bias is to help prepare a person for novel experiences, leading to a different reappraisal of positive memories than of negative memories. For negative memories, the associated emotions and its intensity fade faster than for positive memories which is also known as the fading affect bias (FAB) [26].

Walker and Skowronski [26] speculate that the FAB reflects emotion regulation by dispersing or diminishing the accompanying emotions in negative memories in order for a person not to be distracted, unmotivated, or unprepared for situations that they have to face in the future. Negative memories are thus more likely to decrease in intensity.

Based on these findings, we can infer that differences can exist between how older adults experience the past and present valence of a memory due to a reappraisal of the event, leading to more positively evaluated memories (i.e., positivity bias) and a stronger fading of negative emotions (i.e., fading affect). However, it remains largely unknown whether these experienced differences in emotions can also be found in the way they are expressed.

### Multimodal expressions of emotions

The valence of emotions in expressions can be measured in various ways. One of them is self-reports, which are often used in emotion research [27]. The experienced emotion is described in the content of self-reports as people provide information regarding their pleasure or displeasure (i.e., valence) via self-reports [28,29]. Although the validity of self-reports has been discussed as to whether it is indeed a person’s own felt emotion that is measured and to what extent people are able to describe how they are feeling themselves [27,30], self-reports are important means to take into account the opinion of the person themselves. Furthermore, modalities of emotional expression can be of added value for studying emotions, as research showed that emotional communication is multimodal [31]. Emotions can be expressed in speech, gestures, and facial expressions on which we rely when interacting with each other [32,33]. It is therefore of no surprise that in automatic emotion recognition research, the combination of multiple modalities increase the performance of automatically recognizing emotions [34]. When examining the different emotional expressions of older adults, the added value of having multiple modalities instead of one modality can thus be observed.

Looking at these modalities separately, various studies have researched how valence is expressed in the lexical, acoustic, and facial modality.

#### Lexical modality.

Multiple studies have found that the use of emotional words is associated to self-reports of positive or negative emotions. Tov et al. [35] found that positive emotional words were collected from daily diary entries of participants were related to positive self-reports over time. In addition, negative words were found to be related to self-reports of negative emotions [35]. In a different study, Kahn et al. [36] investigated the relation between positive emotion words and self-reports of amusement after watching comedy films. They found that participants used more positive words after watching the film, and it was related to their self-reports of amusement.

#### Acoustic modality.

Belyk and Brown [37] investigated the acoustic profile of valence in emotion. Participants viewed emotional clips and were instructed to vocalize their response to them in congruent exclamations. A weak association of positive valence with loud vocalizations and high pitch was found. Positive emotions such as happiness were higher in pitch (and amplitude) than negative emotions (e.g., distress). However, other positive emotions such as gratitude were found to be lower in amplitude but still high in pitch whereas pleasure was suggested to be lower in both pitch and amplitude [37]. Although most positive emotions seem to be associated with a higher pitch, it appears that it depends on the context and exclamation of a person, making it difficult to establish a clear relation for pitch and valence. For voice quality, Goudbeek and Scherer [38] found that Hammarberg was positively correlated with valence. In addition, a more varied intensity was associated with positive emotions. Negative emotions were more intense and varied [38]. Schröder et al. [39] found that more pauses (i.e., tempo) and increased intensity were related to negative valenced emotions.

#### Facial modality.

Mortillaro et al. [40] found that certain features in facial expressions were related to positive or negative emotions. Some facial features that are related to smiling (e.g., lip corners) were associated with positive emotions. However, positive emotions were expressed in diverse facial expressions, suggesting that no prototypical facial expression exists for a specific emotion [40]. According to Mortillaro et al. [40], a facial feature cannot isolate one emotion from the other nor can it differentiate between different types of positive emotions such as pleasure and joy. Haines et al. [41] however identified certain features in facial expressions for positive and negative valence. The facial features that represented smiling, such as the dimpler, were related to positive emotions. Other facial features such as the lowering of the eyebrows or wrinkling of the nose were related to more negative emotions such as anger and sadness [41].

Based on previous findings on emotional experiences and expressions in older adults, it becomes clear that the experience of past and present valence in older adults during reminiscence can differ. How the multimodal expressions are related to the past and present valence has yet to be determined. Therefore, the following research questions can thus be formalized:

1. How do older adults’ self-reported past and present valence of personal memories differ from each other?
2. How are self-reported past and present valence related to multimodal expressions of older adults?

For the first research question, a difference between the self-reported past and present valence is expected based on the reappraisal of the memories in accordance to the fading affect bias or the positivity bias. It is hypothesized that older adults will evaluate past memories more positively while the fading affect bias diminishes the intensity of valence for the negative memories. We expect a significant positive difference for sad memories but no significant change is expected for happy memories based on the theoretical rationale presented in the paper.

For the second research question, we expect that both the past and present valence will be more related to the facial, acoustic, and lexical expressions than to only one modality of expressions.

To answer the research questions, we conducted a study with older adults in which they self-reported their past and present valence for emotional memories while their facial, acoustic and lexical expressions were analysed with automatic emotion recognition technology.

## Methods

### Transparency

We report on how we determined our sample size, all data exclusions (if any), all manipulations, and all measures in the study. Preregistration of the study and its analyses does not exist.

### Participants

For the current study, the data are based on 17 participants (sex: 8 female; 9 male) with an age between 66 and 86 years old (M = 74.7, SD = 6.87). Sixteen participants were born in the Netherlands and one participant was born in Indonesia. For education, 10 participants completed higher education, 12 participants completed secondary education, and one participant completed primary education. Participants were recruited through advertisements in local newspapers. The recruitment period started from 20 May 2018 until 31 December 2018. The inclusion criteria for participation were a minimum age of 65 years, fluent reading and speaking of Dutch, and normal or corrected hearing and/or vision. Participants were excluded if they had memory problems, traumatic experiences, or a pacemaker. Inclusion and exclusion criteria were assessed during a phone call with the participants in advance. Data were collected between 7 July and 2 November 2018 from as many participants as possible who applied through the advertisements and met the inclusion criteria requirements. The first author carried out the data collection and the participants were interviewed at their homes.

### Procedure

The Ethics Committee of the University of Twente approved the current study (Nr. 18426). In the submission of ethical approval to the Ethics Committee, we declared that the participants had the capacity to consent. Although the capacity to consent to participate was not assessed as a criterion, the participants consisted of healthy older adults. The participants received the information letter and the informed consent form in advance and were advised to discuss this with their families. Participants were able to ask questions before, during and after the sessions. Participants gave their written consent by signing the informed consent form prior to the study. The study consisted of two sessions of which the current paper focuses on the second session. In the first session, each participant recalled a maximum of three positive and three negative memories via a cue word (i.e., happy or sad). For the second session, these memories were used to create a personal digitalized life story book (LSB) that consisted of short verbal prompts by the participants that represented the happy or sad memory together with a photograph or personal document that the participant shared previously. Often used in reminiscence therapy, LSB increase the quality of life of older adults in geriatric care [42,43]. Here, the LSB of the participant was used to discuss their emotional memories in depth, stimulating conversations, and thereby evoking the positive and negative emotions and expressions of the participants that are related to these memories. The personal LSB was presented on a tablet to the participant. The memories depicted in the LSB were then discussed in depth with the participant. After each memory, the participant rated their past and present valence of this memory. The participants received a small token of gratitude for their participation.

### Experimental design

#### Self-reported past and present valence.

Participants self-rated each memory on a continuous Self-Assessment Manikin (SAM) scale ranging from negative (−1) to positive (+1) [44,45]. The self-reported valence ratings reflect the feelings of the participants regarding their emotional memories in the past and present. After each memory, the participant was asked the question of how they felt when the emotional experience in the memory happened, describing the remembered past valence of the memory. Then, they were asked how they felt when discussing it currently, defining the present valence of the memory in retrospect.

#### Recording set-up.

The recording setup included three video cameras and three microphones. The first video camera captured the frontal view of the participant’s face, whereas the second video camera focused on the interviewer. The third video camera focused on the body of the participant. Two wireless lavalier microphones were used for the participant and interviewer, whereas a shotgun microphone was positioned at the table in front of them. In the current study, only the frontal view video recordings of the participants’ faces and their close-talk recordings were used. A 10.1” tablet was used to show the life story book to the participant and to answer the self-reported past and present valence questions.

#### Transcripts.

The automatic speech recognizer NLSpraak was used to automatically transcribe the memories [46]. They were then manually corrected and anonymised. The transcripts were then segmented based on their thematic coherence. Thus, a memory could be divided into several meaningful topic segments, i.e., an emotional memory of a divorce could be chunked into the segment of infidelity, divorce, and financial troubles. In total, 625 topic segments were established from 95 emotional memories of 17 participants.

#### Feature extraction.

For the multimodal expressions in older adults, recognition technology was used to automatically extract multiple features from the lexical, acoustic and facial modalities.

#### Lexical features.

For the lexical expressions, sentiment analyses were performed on the topic segments based on the Pattern library [47] to establish the polarity (i.e., classification of negative, neutral of positive valence) of the transcripts of the memories. Consisting of Dutch words that have polarity strengths and intensity values, Pattern is an open source lexicon with an algorithm that accounts for negations, downtoners, and intensifiers. Negations focus on distinguishing between “not angry”, “really not angry” or “not really angry”. A sentiment of an adjective can be diminished or strengthened based on the adverb used, which is known as downtoners (i.e., “terribly beautiful”). Additionally, the consisting Dutch lexicon was broadened by including Dutch affective words by Moors et al. [48]. Affective words with a neutral valence score were excluded from the lexicon, as the focus of the current study was only on affective words contributing to the emotional expressions through valence. For each sentence within a segment, the mean and standard deviation of the sentiment scores were calculated and averaged for a segment. This resulted in sentiment scores that ranged from negative (−1) to positive (+1) valence.

#### Acoustic features.

Consistent with our previous research [49], the same acoustic features were selected for the current study (see Table 1). These features were expected to be minimally correlated with each other and associated with valence. Praat [50] was used to extract the acoustic features. For F_0_ and intensity, the mean, standard deviation, and range were extracted. In addition, voice quality (spectral balance), tempo information, and the Hammarberg Index (dB) [51] were added. Indicating the distribution of energy in the spectrum, the Hammarberg Index is characterized as the difference between the maximum energy in the higher (2000-5000Hz) and lower (0-2000Hz) frequency bands [49]. A script by de Jong et al. [52] was used to extract the articulation rate by defining the amount of syllables per second without silences. The amount of silences per second defined the pause rate. By manually setting a threshold for intensity in Praat (i.e., minimum duration of sound 150ms, minimum duration of silence 500ms), the silent parts were identified. The silent parts were discarded for the feature extraction for F_0_, intensity, voice quality, and articulation rate. The acoustic features for each speaker were then standardized by transforming them into z-scores.

**Table 1:** Acoustic features used in the study.

Acoustic	Features
Pitch	mean, standard deviation, range of F_0_
Intensity	mean, standard deviation, range
Voice quality	Hammarberg Index [51]
Tempo	mean pause duration, articulation rate, pause rate

#### Facial features.

The facial features were analyzed with OpenFace 2.0 [53], which employs a number of models to perform facial detection and landmark tracking. In addition, detection of the intensity and presence of a facial action unit (AU) [54] is performed. A selection of AUs is obtained for each video frame and these are subsequently filtered and aggregated per topic segment. In addition to the mean and standard deviation, the normalized duration for which an AU is active is also calculated. An AU is considered to be active for a time-span if the value remains above a threshold (50% of the value range) for longer than 0.1 second. The duration of these spans are summed and normalized based on the duration of the corresponding topic segment. For the current paper, a selection of AUs and their intensity was used. This selection was based on the AUs that were found to be associated with positive and negative emotions according to the literature [41,55–61]. The authors then interpreted the AUs on the valence dimension based on this association. Given that some AUs can be related to both positive and negative emotions, depending on their combinations with other AUs for an emotional expression, valence was categorized as either “positive”, “negative” or “positive or negative”. Table 2 shows an overview of the facial features and the association with valence used in the current study. This selection of AUs was also the least affected by our experimental setting, reducing the false error rate.

**Table 2:** Facial features used in the study.

AU	Feature	Description	Valence
AU01	mean, standard deviation, duration per minute	Inner Brow Raiser	Positive or Negative
AU04	mean, standard deviation, duration per minute	Brow Lowerer	Negative
AU09	mean, standard deviation, duration per minute	Nose Wrinkler	Negative
AU10	mean, standard deviation, duration per minute	Upper Lip Raiser	Negative
AU12	mean, standard deviation, duration per minute	Lip Corner Puller	Positive
AU14	mean, standard deviation, duration per minute	Dimpler	Positive or Negative
AU15	mean, standard deviation, duration per minute	Lip Corner Depressor	Negative

#### Statistical analysis.

First, two paired-sample t-tests were conducted to compare the self-reported past and present valence for the happy and sad memories. Past valence was used as a pre-test score whereas present valence was used as a post-test score. To explore the relation of lexical, acoustic, and facial features with the past and present valence in emotional memories of older adults, linear mixed-effects model analyses were used to compare the different predictors of the two self-reported valence scores (as dependent variables). In addition, the control variables age, gender, and cue word (i.e., “happy” or “sad” that was used to elicit the positive or negative memory) also acted as predictors, whereas participant acted as the random effect. Predictors were then excluded stepwise based on the removal of the predictor with the highest non-significant p-value first to improve the model fit. The Akaike Information Criteria (AIC) was chosen to select the model with the most parsimonious fit. This resulted in the best-fitting multimodal models of past and present valence including all lexical, acoustic, and facial features. To compare the multimodal models to their unimodal models, the lexical-only, acoustic-only, and facial-only models for past and present valence were also created in the same manner. The statistical software R, version 1.3.1093 [62] was used to compute the linear mixed-effect model analyses with the packages lme4, version 1.1-23 [63], lmertest, version 3.1-2 [64] and sjPlot, version 2.8.5 [65]. The marginal *R*^2^ of the mixed-effect model was computed with the package piecewiseSEM, version 2.1.0 [66]. The package ggplot, version 3.3.-6 [67] was used to plot the data. Multicollinearity was conducted with the package performance, version 0.14.0 [68].

## Results

### Comparison of the self-reported past and present valence

**Fig 1:**
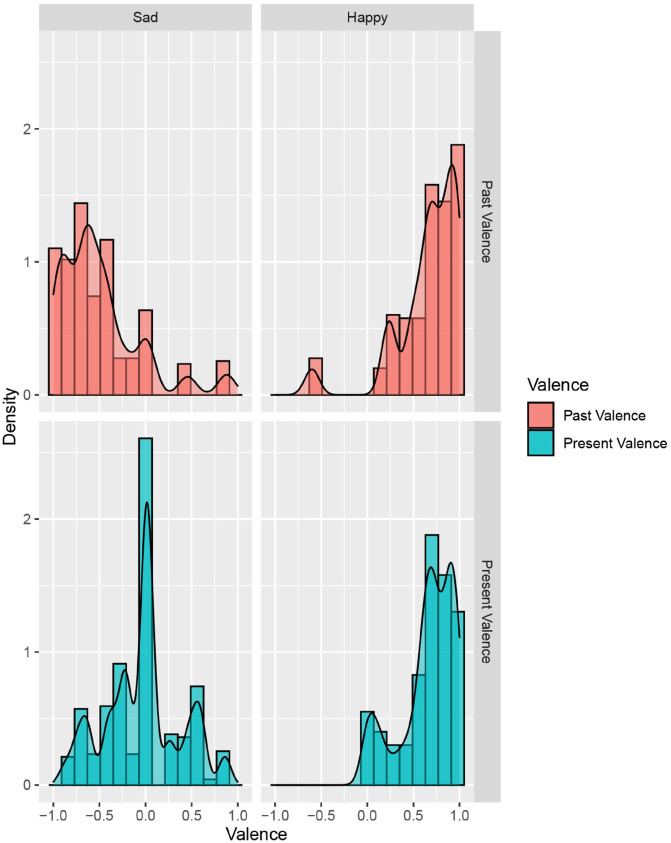
Histogram and density plots of the scores of the self-reported past and present valence divided by the cue words (“happy” and “sad”) for the emotional memories.

For the first research question of how older adults’ self-reported past and present valence of personal memories differ from each other, Fig 1 shows the scores of the past and present valence categorized according to the sad or happy memories. A bimodal distribution can be seen in the scores of past valence, suggesting that older adults rated their memories mainly either negative (−1) or positive (+1) on the valence scale. This means that they felt either largely negative or positive about their sad or happy memories when the event happened. Sad memories were indeed evaluated more negatively, whereas happy memories were predominantly evaluated more positively by older adults. However, the scores of present valence shifted toward the neutral midpoint. Similarly to past valence, the happy memories were rated positive for present valence. However, older adults scored their sad memories more in the middle of the valence scale. Their current feelings regarding their past sad memories were thus less negative than when it happened in the past. The two paired-sample t-tests indeed showed a significant difference between the past and present valence for the happy memories as well as the sad memories. For the happy memories, scores for the present valence were significantly higher than the scores for the past valence (M = .078, SD = .38, *t*(306) = 3.605, *p* < .001, *d* = .206) despite the low magnitude of means difference. For the sad memories, scores for the present valence were significantly higher than the scores for the past valence (M = .412, SD = .471, *t*(314) = 15.537, *p* < .001, *d* = .875). These results offer support to the hypothesis that states that there is a difference in how older adults rate their past and present valence with respect to their personal memories.

### Multimodal expressions of past and present valence

To answer the second research question of how self-reported past and present valence are related to multimodal expressions, Tables 3 and 4 show that the explained variance for the best-fitting multimodal past valence model is *R*^2^ = 0.463. For the best-fitting multimodal present valence model, we found the explained variance to be *R*^2^ = 0.387. To check for multicollinearity, Variance Inflation Factors (VIF) were calculated for the predictors in the best-fitting past and present multimodal models. The VIF factors for all predictors in the past and present models were below 5, indicating low multicollinearity [69]. The small difference of 7% explained variance between the two models suggests a small effect that the past valence model seems slightly more related to the multimodal expressions.

**Table 3:** Model comparison of the best-fitting multimodal model, acoustic only, lexical only and facial only model of the self-reported past valence model.

Model	AIC	marginal *R*^2^
Multimodal model	886.48	0.463
Lexical only	969.00	0.368
Acoustic only	978.31	0.359
Facial only	948.87	0.419

**Table 4:** Model comparison of the best-fitting multimodal model, acoustic only, lexical only and facial only model of the self-reported present valence model.

Model	AIC	marginal *R*^2^
Multimodal model	525.72	0.387
Lexical only	572.67	0.255
Acoustic only	582.94	0.248
Facial only	557.94	0.364

Focusing on the comparison between the multimodal past valence model and the unimodal models, Table 3 shows that the multimodal model explained the most variance (*R*^2^ = 0.463). Then, the facial-only model (*R*^2^ = 0.419) contributed to a larger share of the total variance than the acoustic-only (*R*^2^ = 0.359) and lexical-only (*R*^2^ = 0.368) models.

For the present valence, the multimodal model accounted for most of the variance (*R*^2^ = 0.387), as shown in Table 4. The facial-only model (*R*^2^ = 0.364) explained more of the total variance than the acoustic-only model (*R*^2^ = 0.248) and the lexical-only model (*R*^2^ = 0.255). Both the multimodal past and present valence models show that the multimodal models work better than the unimodal models.

For both the multimodal models, the multimodal features combined were more strongly related to valence than the lexical, acoustic or facial only modalities. This shows that a multimodal model gives more information about the past and present valence than only one modality. When looking at the features in both the past and present multimodal models, it appears that the facial features have the strongest association with past and present valence. Thereafter, the acoustic features add less predictive value, and the lexical features are least represented in both models.

#### Control variable.

The control variable Cue Word “happy” acted as the intercept. Cue Word was found to be significant for both past and present valence. The hypotheses were confirmed that happy memories had a similar valence in the past and present whereas the valence of sad memories was reappraised more positively in the present than in the past (Tables 5 and 6). Age and gender were not significant for the past and present valence.

**Table 5:** Estimates of the fixed effects of the best-fitting model of the self-reported past valence; AIC = 886.48 with 622 observations (df = 603). Marginal *R*^2^ = 0.463.

Feature	β	SE	CI	p*
Cue word	−0.70	0.04	−0.78 – −0.62	**<0.001**
Sentiment mean	0.70	0.11	0.48 – 0.93	**<0.001**
Sentiment variance	−0.30	0.15	−0.59 – −0.00	**0.046**
*F*_0_ mean	0.04	0.02	0.00 – 0.08	**0.047**
Duration pause mean	−0.09	0.02	−0.13 – −0.05	**<0.001**
Hammarberg Index	−0.06	0.02	−0.09 – −0.02	**0.004**
AU01 mean	0.03	0.01	0.01 – 0.06	**0.007**
AU01 duration	0.01	0.00	0.00 – 0.01	**0.042**
AU04 duration	−0.00	0.00	−0.01 – −0.00	**0.031**
AU10 duration	0.01	0.00	0.00 – 0.01	**0.003**
AU12 duration	0.01	0.00	0.01 – 0.02	**<0.001**
AU15 variance	0.30	0.10	0.10 – 0.51	**0.004**

*Statistical significance is indicated in bold face.

**Table 6:** Estimates of the fixed effects of the best-fitting model of the self-reported present valence data; AIC = 463.81 with 622 observations (df = 604). Marginal *R*^2^ = 0.281.

Feature	β	SE	CI	p*
Cue word	−0.41	0.03	−0.47 – −0.35	**<0.001**
Sentiment mean	0.38	0.08	0.21 – 0.54	**<0.001**
*F*_0_ mean	0.03	0.01	0.00 – 0.06	**0.032**
Duration pause mean	−0.05	0.01	−0.08 – −0.02	**<0.001**
AU01 mean	0.02	0.01	0.00 – 0.03	**0.047**
AU01 duration	0.01	0.00	0.00 – 0.01	**0.004**
AU09 duration	−0.01	0.01	−0.02 – −0.00	**0.024**
AU10 duration	0.01	0.00	0.00 – 0.01	**<0.001**

*Statistical significance is indicated in bold face.

#### Multimodal features.

The estimates of the significant features of the past and present valence models are shown in Tables 5 and 6.

#### Lexical features.

For both past and present valence, sentiment was found to be significant, which means that the use of more positive words when recalling the memory is related to a more positive feeling of the participant during that memory at that time and in the present. The variance in sentiment was found to be significant (p <.046) for past valence only, showing a negative association with higher valence, indicating that the use of positive words when recalling the memories varied more when the participant’s feeling during that event in the past was more negative.

#### Acoustic features.

The duration of the pauses was found to be significant for both past and present valence, showing that longer pauses in the memories were related to a more negative feeling of the older adult during the event and in retrospect. Thus, older adults had shorter pauses in the memories that were related to more positive feelings when the memory happened and while discussing it now. In addition, F0 mean was positively associated with both past and present valence, meaning that a higher pitch in discussing the memories was related to a more positive reported feeling of the older adults at the time the memory happened and in the present. Lastly, the Hammarberg Index was found significant for past valence, suggesting that a lower Hammarberg Index was associated with a more positive feeling reported by the older adults. This means that a flatter spectral slope and relatively more energy in the higher frequencies are related to a more positive feeling and vocal effort of the older adults.

#### Facial features.

The intensity and duration of AU01 (inner brow raiser) were significant for both past and present valence, which were positively associated with higher valence. Older adults had a more positive feeling in the past and present when recalling emotional memories, as the inner brow was more intensively and longer raised in the face. The duration of AU04 (brow lowerer) was negatively related to a higher past valence, showing that a shorter lowering of the brow was related to a more positive feeling of older adults. A negative association was found for the duration of AU09 (nose wrinkler) and present valence, meaning that a longer wrinkling of the nose was related to a more negative feeling about their emotional memory in retrospect. For both past and present valence, the duration of AU10 (upper lip raiser) was found to be significant. A longer raise of the upper lip was associated with a more positive past and current feeling about the memory. AU12 (lip corner puller) was positively associated with higher past valence, suggesting that a longer pull of the lip was associated to a more positive feeling of the participant when the event happened. Finally, the variance in the intensity of AU15 (lip corner depressor) was positively associated with past valence. This implies that more variability was seen in the depressors of the lip corners of the participants when they were feeling more positive when the event happened.

## Discussion

The goal of this paper was to determine how the self-reported past and present valence of memories differed from each other and how it was related to the multimodal expressions of older adults. For the first research question, a difference in valence was observed for the personal memories. Older adults felt more positive about their happy memories in the present than in the past. A change in valence was more apparent for the sad memories as the valence showed a significant reduction in negativity when comparing the past and present valence of sad memories. The difference from past to present valence is larger for negative memories than for positive memories. This is due to the reappraisal of the negative memory, in which the fading affect and positivity bias could be an explanation for the change in valence. As previously described, Kaplan, Levine, and Safer [14] argued that people would base their emotions on the current appraisal of the importance of the goal to the event. The fading affect and positivity bias are related to a person’s current goal, which alters the reappraisal of the event in the present and results in less intense negative emotions for negative memories. Our findings showed that the past negative emotions are now more faded and diminished over time, which suggests that a fading affect is in place. As stated earlier, the FAB describes that the associated emotions in negative memories fade faster than for positive memories [26], therefore resulting in a more neutral present valence, as our findings show. Concurrently, the positivity bias could also play a role in the present valence. Although present valence was not positively rated for negative memories, it was evaluated as less negative as compared to the past. This illustrates a slight positive bias in remembering the past more positively and ignoring negative emotions [22,70]. However, it remains difficult to determine which bias is responsible for this shift in valence. Nonetheless, it does unveil that when measuring emotions, sad memories would not necessarily result in negative valence.

For the second research question, the multimodal models work better in explaining the past and present values than the models with only one modality, as hypothesized. However, the results were more complex than that. It appears that the past valence is related slightly stronger to multimodal expressions than the present valence, given the somewhat larger explained variance. Some features were only related to past valence, others were related to present valence while some features were relevant to both past and present valence.

For past valence, the voice quality, lowering of the brow and the lip corners are more important. We found that a longer pull of the lip corner relates to a more positive feeling while a longer brow lowering related to a more negative past feeling. In line with our findings, previous studies indeed showed that lip corner pullers and brow lowering are often associated with positive and negative emotions [40,41,57]. For voice quality, a higher past valence was related to a lower Hammarberg Index, meaning that if the spectral slope was less steep, a more positive feeling in the past was reported. A higher vocal effort (i.e., more energy in the higher frequency band) was related to a higher positive past valence. As described earlier, Goudbeek and Scherer [38] found that the Hammarberg Index was positively correlated (i.e., steeper spectral slope) with valence, which contradicts our findings. A possible explanation could be that Goudbeek and Scherer [38] studied actors who expressed positive and negative emotions, while our study assessed older adults who recall their emotional memories. In addition, eliciting happy and sad memories could result in differences not only in valence but in arousal as well. According to some studies, flatter spectral slopes are related to high arousal [39,71]. As arousal or emotional intensity was not examined nor controlled for in the current study, this could possibly explain the low Hammarberg Index for valence. Interestingly, our previous research in older adults telling memories found similar results to the current findings [49].

For the present valence, the wrinkling of the nose matters more as it relates to current negative feelings. In earlier research, nose wrinkling is often associated with negative emotions [60,72–74]. In a study by Haines et al. [41], the wrinkling of the nose was related to more negative emotions such as anger and sadness. These studies support our findings, although no distinction between past and present valence has been made as in our study.

Lastly, there are also features that are important for both past and present valence. The tempo that older adults have in their speech, pitch height, their word use, the raising of the inner brow, and upper lip were found to be related to both past and present valence. These findings for the specific modalities were in general in line with previous research that has shown that older adults talk in a slower tempo for negative felt memories and they use more positive and less negative words when describing their positive felt memories [6,35,39,49]. Furthermore, research showed that the inner brow raiser is both present in positive and negative emotions [72,75], while we found a positive relation between the (longer) raising of the inner brow and valence. Our study used emotional memory recall while the other studies used either film clips or consumer product-based stimuli, which could explain the difference. Pitch height was found to be positively related to past and present valence which is in line with other research [76]. Interestingly, our previous study found a negative association between valence and pitch [49]. However, the valence in the previous study was annotated on topic segmentation, while the current study used self-reported valence, which could explain the difference. As previously discussed, the relation between valence and pitch can be unclear [37,77] as pitch heights can vary for different positive emotions. Happiness can have a high pitch, whereas pleasure can have a low pitch which explains the different pitch heights for positive emotions. The current study focused only on the valence dimension, making it therefore difficult to distinguish between different positive emotions.

However, there are also some findings in the past and present valence that are not in line with previous research. According to other research, a longer upper lip raiser and variability in the lip corner depressor are often related to negative emotions [75,78]. A possible explanation for this contradiction could be the talking by the older adults during memory recall. To examine the effect of talking, two post-hoc analyses were performed for past and present valence with AUs that were now only extracted when participants were silent, thereby eliminating the influence of talking. The post-hoc analyses for both past and present valence showed inconclusive evidence of the influence of talking. Some AUs still remained significant, whereas others were excluded inconsistently. Due to these ambiguous results, the effect of talking remains unclear. Caution must therefore be taken when interpreting the current findings.

In all, voice quality, lowering of the brow and the lip corners are relevant for past valence. For present valence, the wrinkling of the nose is more relevant. The tempo, word use, pitch height, the raising of the inner brow and the raising of the upper lip are important for both past and present valence.

## Limitations and future research

### Limitations

Several limitations were present for the current study. One of the limitations was the single dimension for the emotional experience, namely self-reported valence. As no other dimensions were assessed in this study, it is not possible to discriminate between different emotional states. This limited our interpretations of some modalities, such as the acoustic and facial features, which are often measured in arousal or emotional intensity. Secondly, the accuracy of automatic emotion recognition technology can be discussed. Previous research found that OpenFace performed poorly in identifying AUs on data from a real-world environment in comparison to trained human facial coders [79]. OpenFace is trained on controlled environment data, which could explain the difference. Additionally, age-related changes could also have impacted the performance of the automatic emotion recognition technology. Folds and wrinkles can resemble emotions (i.e., wrinkles around the eye are due to frowning or smiling) and can be mistakenly identified as emotions even in their absence [1,80]. Eyesight also deteriorates with age, resulting in more older adults wearing glasses [81]. This in turn could have obstructed the automatic recognition of the facial features in the eye region and brow, making it more difficult to correctly identify these regions [82,83]. Research indeed found that OpenFace performed worse when AUs were obstructed by glasses [84]. As our data are collected in real world environments with participants who wear glasses, there is a possibility that the accuracy for detecting correctly the AUs is lower. Nevertheless, significant relations between facial expressions and self-reported valence were found. Lastly, the facial feature extraction could be affected by facial movements that are due to talking and not due to emotions per se. Although our experimental design resulted in spontaneous emotions and expressive behavior, such as crying and laughing during reminiscence, the findings for the facial features have to be interpreted with caution due to the possible impact of talking.

### Future research

It appears that the reappraisal of the memories by older adults largely explained the current findings in past and present valence. It raises then the question with what purpose people reappraise their emotional memories. Studies have shown that people use different functions of reminiscence to tell their story [85–87]. It would be interesting to examine the goal of reminiscence by older adults to gain insight into the reappraisal process and how it is related to the emotional experiences and expressions. Earlier research identified different functions of reminiscence, for example integrative reminiscence [86,88]. Integrative reminiscence is focused on the integration of the past and present and on solving and reconciling conflicts [89]. It has as function to accept and resolve past negative events. In a way, people are reappraising their past constructively and integrating it into the present [87]. Perhaps with age, older adults are more reminded of how they reconciled, resolved, accepted, or coped with negative events in the past, resulting in a different appraisal of these events in the present. As reminiscence function was not controlled for in the current study, older adults could have applied different types of reminiscence during memory recall. The relation between reminiscence type and emotional experience and expressions would therefore be interesting to examine in future research. Lastly, the current study is based on 17 older adults. A comparison group such as younger adults to better understand and identify the impact of aging on valence, memories, and multimodal expression would be recommended. As life experiences increase with age, one can wonder whether the same findings will be found in younger adults.

## Conclusion

When studying emotions in memories, it is important to distinguish between the past and present valence of older adults’ memories. The expressions of emotions are more strongly related to the past valence due to reappraisal, which is interesting as it provides a better understanding of the emotional valence in older adults. This allows us to better support their needs by focusing on their past and present feelings of emotional memories to eventually improve their quality of life and well-being. Although caution must be taken when interpreting the findings of the different modalities, further research can benefit the development of automatic emotion recognition technology for older adults in the future. This in turn can assist them in their needs and increase the understanding of emotions for this under-represented group.
